# E4BP4 promotes thyroid cancer proliferation by modulating iron homeostasis through repression of hepcidin

**DOI:** 10.1038/s41419-018-1001-3

**Published:** 2018-09-24

**Authors:** Qinyi Zhou, Jun Chen, Jialin Feng, Jiadong Wang

**Affiliations:** Department of Head and Neck Surgery, Renji Hospital, School of Medicine, Shanghai Jiaotong University, Shanghai, China

## Abstract

Iron homeostasis is critical to mammals, and dysregulation in iron homeostasis usually leads to severe disorders including various cancers. Massive hepcidin secretion is an indicator of thyroid cancer, but the molecular mechanisms responsible for this dysregulation are unknown. Hepcidin secretion from thyroid cancer cells also leads to decreased expression of the iron exporter, ferroportin (FPN), and increased intracellular iron retention, which promote cancer proliferation. In this study, we examined the role of hepcidin in thyroid cancer (TC) and the molecular bases of its signaling. Synthesis of hepcidin is regulated by the BMP4/7 agonist SOSTDC1, which was downregulated in TC; SOSTDC1 downregulation was correlated with G9a-mediated hypermethylation in its promoter. The binding of G9a to the SOSTDC1 promoter requires E4BP4, which interacts with G9a to form a multi-molecular complex that contributes to SOSTDC1 silencing. Silencing of E4BP4 or G9a has similar effects to SOSTDC1 overexpression, which suppresses secretion of hepcidin and inhibits TC cell proliferation. Furthermore, our in vivo xenograft data indicated that depletion of E4BP4 also inhibits cancer growth, reduces hepcidin secretion, and reduces G9a nuclear transportation. Iron homeostasis and tumor growth in TC may be regulated by an E4BP4-dependent epigenetic mechanism. These findings suggest a new mechanism of cellular iron dysfunction through the E4BP4/G9a/SOSTDC1/hepcidin pathway, which is an essential link in TC.

## Introduction

Thyroid cancer (TC) is one of the frequent malignancies of the endocrine system, with a high incidence rate^1^. Histologically, it can be divided into three subtypes, including differentiated papillary carcinoma, follicular carcinoma, and undifferentiated anaplastic carcinoma. It has been reported that genetic and epigenetic modifications are involved in TC^1,2^. Therefore, there is a pressing need to determine the genetic factors contributing to TC.

Iron homeostasis is critical for biological processes in normal cells^3^, and disruption of iron homeostasis causes various cellular disorders such as growth arrest; excessive iron can damage proteins, DNA, and other cell constituents^3,4^. Furthermore, recent studies have confirmed the indispensable role of iron in growth of cancer cell^5^. It has been shown that uptake, storage, and discharge of iron are altered in cancer cells, which facilitate their survival^5,6^. Therefore, molecules that regulate iron metabolism are potential therapeutic targets. The protein hepcidin is delivered to the specific tissues through the circulation^7^. In the duodenum, hepcidin can curb absorption of irons, while in macrophages and hepatocytes, it promotes cellular retention of iron by triggering degradation of ferroportin (FPN)^8^. Increased serum hepcidin level serves as an indicator of various cancers, including myeloma^9–12^. Autocrine of hepcidin and expression of its receptor FPN are also found in tumors^11,12^. Moreover, increased FPN expression level is correlated with high survival rate in cancer patients^11,13^. However, the molecular mechanisms responsible for dysregulation of hepcidin in TC are still unknown.

As a central regulator of hepcidin levels in prostatic cells, SOSTDC1 can inhibit both the BMP and Wnt signaling pathways^12^. Studies have shown that silencing of SOSTDC1 is correlated with cancer progression^12^. Moreover, SOSTDC1 is involved in cell signaling pathways that regulate normal embryonic development and cancer^14^. Of note, expression of SOSTDC1 in cells vary with different cell cycle status^15^, and when suppressed, SOSTDC1 can accelerate tumor development and progression. Studies in gastric cancer revealed that SOSTDC1 is regulated through epigenetic modification, and that it is downregulated via promoter hypermethylation^16^, which leads to increased secretion of hepcidin in prostate cancer^12^. However, the role of SOSTDC1 in TC as well as the mechanisms underlying promoter hypermethylation remains unknown.

In this study, we aimed to elucidate the function of hepcidin in TCs and the molecular basis of its signaling. We found that compared with that in controls, hepcidin secretion is higher in TC patients, as well as TC cell lines. Results indicated that SOSTDC1 silencing via promoter hypermethylation contributes to hepcidin secretion in TC. Furthermore, hypermethylation of the SOSTDC1 promoter is regulated by the E4BP4 and G9a complex. These data revealed a potential correlation between TC and iron homeostasis, which can provide theoretical evidence for TC.

## Results

### Hepcidin is upregulated and downregulates FPN in TC cells

**Fig. 1:**
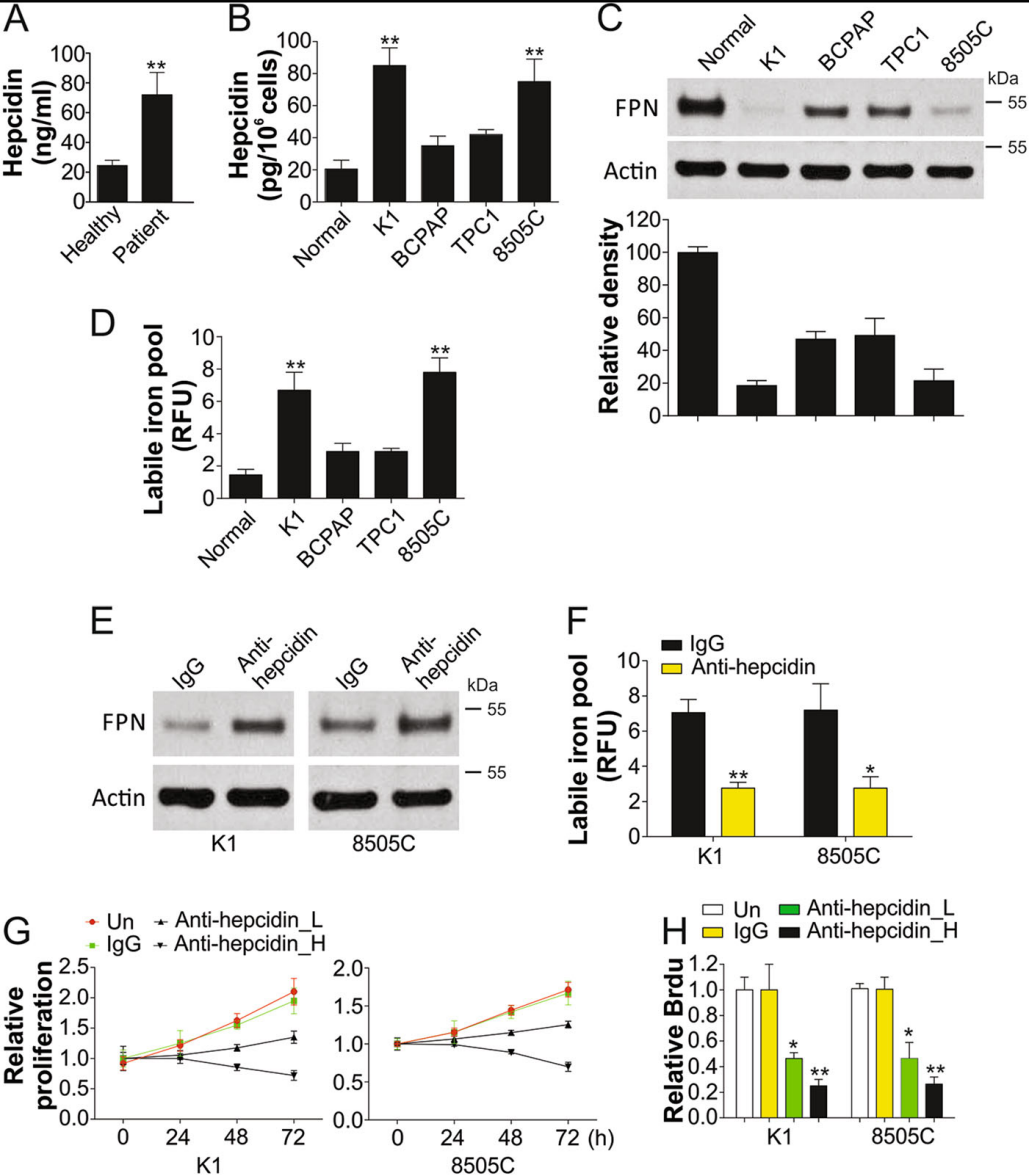
Hepcidin is upregulated in TC cells and modulates the levels of iron. **a** The secretion of hepcidin in TC patients (*n* = 48) and healthy participants (*n* = 38). **b** The secretion of hepcidin in the Nthy-ori 3-1 cells and TC cells (K1, BCPAP, TPC1, and 8505C). **c** Upper, the western blot of ferroportin (FPN) in indicated thyroid cells; lower, the relative density of western blot was analyzed by Image J software. **d** The intracellular labile iron contents in indicated thyroid cells. **e** The expression of FPN in K1 and 8505C cells after treatment of control antibody or antibody of hepcidin (3 µg/ml). **f** The intracellular labile iron contents in K1 and 8505C cells after treatment of control antibody or antibody of hepcidin (3 µg/ml). **g** The proliferation of K1 and 8505C cells after treatment of control antibody or antibody of hepcidin (2 µg/ml, L; 3 µg/ml, **h**) was analyzed by MTT assay. **h** BrdU analysis of K1 and 8505C cells proliferation after treatment of control antibody or antibody of hepcidin (2 µg/ml, L; 3 µg/ml, **h**). Each experiment was repeated for three times. *P < 0.05, **P < 0.01

To investigate the correlation of hepcidin expression level in TC, we compared the serum hepcidin levels in TC patients and age-matched healthy participants. We found that hepcidin secretion level was significantly higher in TC patients than in healthy subjects (Fig. 1a). This suggested that increased serum hepcidin level is positively correlated with TC.

We further explored differences in hepcidin expression through in vitro experiments; and found that the hepcidin was increased obviously in TC cells, especially in K1 and 8505C cells, which was significantly higher than that in normal cells (Fig. 1b). Next, we focused on pathways leading to intracellular increase in iron; hepcidin and FPN levels were higher and lower in thyroid cells, respectively, than in normal cells (Fig. 1c). Intracellular iron content in TC cells was higher than that in non-malignant thyroid epithelial cells (Fig. 1d), especially in cell lines exhibiting higher hepcidin secretion, such as K1 and 8505c cells. To determine how hepcidin regulates FPN, we treated TC cells with anti-hepcidin antibodies. We found that antibody-mediated blockade of hepcidin increased FPN in K1 and 8505C cells (Fig. 1e), but reduced intracellular iron content in K1 and 8505C cells (Fig. 1f). Accordingly, inhibition of hepcidin by its antibody also and inhibited cell proliferation in a dosage-dependent manner (Fig. 1g, h). Thus, TC cells can synthesize functional hepcidin to promote TC cell proliferation.

### SOSTDC1 regulates hepcidin synthesis in TC cells by inhibiting BMP4/7

**Fig. 2:**
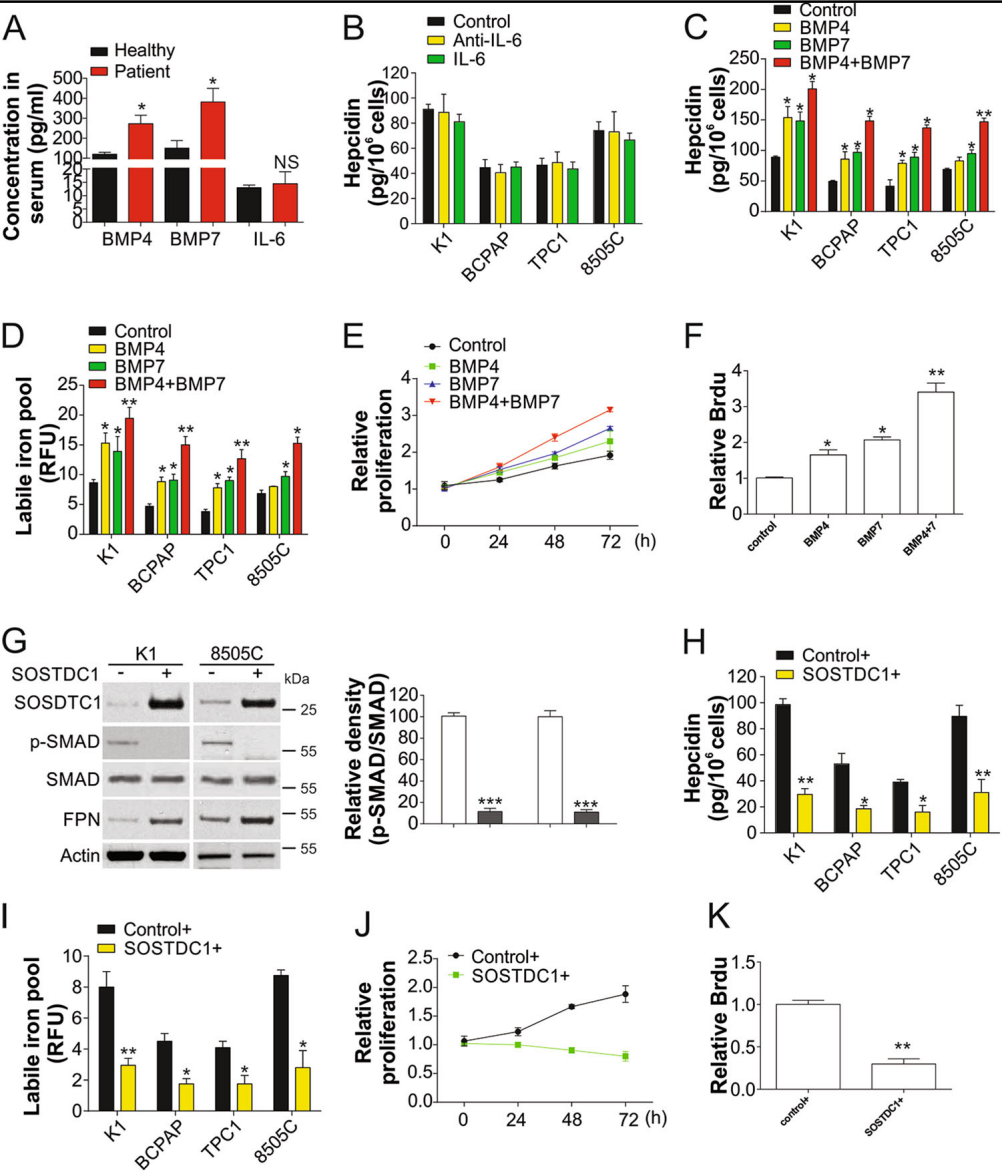
Hepcidin secretion in TC cells is modulated by SOSTDC1. **a** The concentration of BMP4, BMP7, IL-6 in the serum of TC patients (*n* = 48) and healthy participants (*n* = 38). **b** The secretion of hepcidin in the indicated TC cells treated with antibodies of IL-6 or IL-6 for 24 h. **c** The secretion of hepcidin in the indicated TC cells treated with BMP4, BMP7, or their combination for 24 h. **d** The intracellular labile iron contents in indicated TC cells treated as in (**c**). **e** Proliferation of cells treated in (**c**) was analyzed by MTT assay. **f** Proliferation of cells treated in (**c**) was analyzed by BrdU assay. **g** Left, protein expressions in K1 and 8505C cells transfected by SOSTDC1 plasmid; right, the relative density of p-SMAD vs. SMAD was analyzed by Image J software. **h** The secretion of hepcidin in indicated TC cells transfected with SOSTDC1 plasmid. **i** The intracellular labile iron contents in in indicated TC cells transfected with SOSTDC1 plasmid. **j** The proliferation of K1 cells transfected with SOSTDC1 plasmid was analyzed by MTT assay. **k** The proliferation of K1 cells transfected with SOSTDC1 plasmid was analyzed by BrdU assay. Each experiment was repeated for three times. NS *P* > 0.05, *P < 0.05, **P < 0.01

Since IL-6 and BMPs are well known regulators of hepcidin^12^, we firstly analyzed serum IL-6, BMP4, and BMP7 levels by ELISA, and found that secretion of only BMP4 and BMP7 was increased in TC patients (Fig. 2a). This suggested that BMP4/7 were the major contributors to hepcidin secretion in TC. The addition of IL-6 or anti-IL-6 antibody did not significantly affect hepcidin secretion (Fig. 2b), further indicating that IL-6 is not associated with elevated hepcidin levels in TC cells. Previous studies have also shown that BMP4 and BMP7 are critical to expression of hepcidin in the liver^12^. The combination of BMP4 and BMP7 induced higher hepcidin secretion from TC cells than single treatment alone (Fig. 2c). Furthermore, BMP4 and BMP7 together also maximally increased intracellular iron contents in TC cells (Fig. 2d) and promoted cell proliferation (Fig. 2e, f), suggesting that BMP4 and BMP7 mediate hepcidin secretion by TC cells.

BMP is mainly regulated by its antagonists^19^, particularly SOSTDC1, which can bind to and inhibit BMP2, BMP4, and BMP7^12^. Therefore, we investigated whether enhanced expression of this antagonist could suppress hepcidin secretion by TC cells. Overexpression of SOSTDC1 in TC cells resulted in attenuation of SMAD phosphorylation, which is mediated by BMP4/7 signaling, and increased the expression of FPN (Fig. 2g). Enhanced SOSTDC1 expression also suppressed hepcidin secretion (Fig. 2h), intracellular iron content (Fig. 2i), and cell proliferation (Fig. 2j, k). Collectively, these results indicate that SOSTDC1 regulates secretion of hepcidin by TC cells via inhibition of BMP4/7.

### SOSTDC1 is downregulated by promoter methylation

**Fig. 3:**
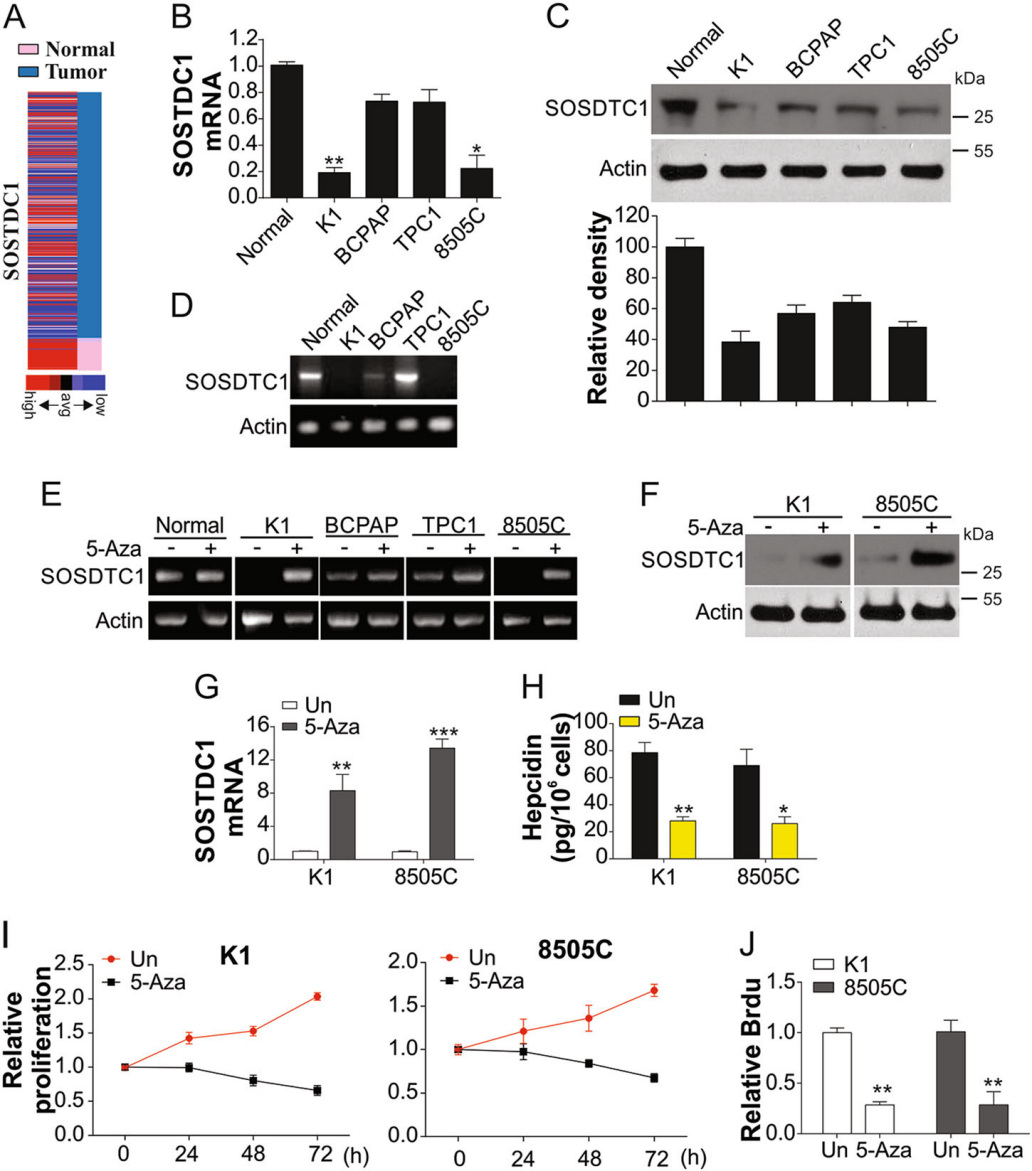
The methylation of SOSTDC1 promoter suppressed its expression. **a** SOSTC1 expression in TC from TCGA database. **b** mRNA levels of SOSTDC1 in indicated cell lines. **c** Upper, protein levels of SOSTDC1 in indicated cell lines; lower, the relative density was analyzed by image J software. **d** Bisulfite-modified genomic DNA without promoter hypermethylation of SOSTDC1 was analyzed by methylation-specific PCR. **e** The genomic DNA from indicated cells with or without 5-Aza. **f** Protein expressions of SOSTDC1 in TC cells with or without 5-Aza. **g** mRNA expression of SOSTDC1 in TC cells with or without 5-Aza. **h** The secretion of hepcidin in TC cells with or without 5-Aza. **i** The proliferation TC cells with or without 5-Aza was analyzed by MTT assay. **j** The proliferation TC cells with or without 5-Aza was analyzed by BrdU assay. Each experiment was repeated for three times. *P < 0.05, **P < 0.01, ***P < 0.001

By analyzing the mRNA expression of SOSTDC1 in the TGCA database (https://genome-cancer.ucsc.edu/proj/site/hgHeatmap/), we found that SOSTDC1 is silenced in most TCs (Fig. 3a), as confirmed by previous studies^12,20^. We next measured the expression of SOSTDC1 in thyroid cells, and found that SOSTDC1 expression was dramatically reduced, especially in K1 and 8505C cells (Fig. 3b, c). We hypothesized that this phenomenon may be due to DNA methylation, which is a major contributor to gene silencing in cancers^21^. As shown in Fig. 3d, DNA methylation was observed in K1 and 8505C cancer cells, which exhibited low SOSTDC1 expression. Our results indicated that in the presence of 5′-aza-dC, SOSTDC1 promoter methylation in K1 and 8505C cells was significantly reduced (Fig. 3e). SOSTDC1 expression was also recovered in 5′-aza-dC treated K1 and 8505C cells (Fig. 3f, g). We found that recovery of SOSTDC1 by 5-aza-dC led to suppression of hepcidin expression (Fig. 3h) and cell proliferation (Fig. 3i, j). Overall, these data indicated the methylation of SOSTDC1 promoter leads to downregulation of SOSTDC1, and consequently results in hepcidin secretion by TC cells.

### G9a mediates promoter methylation of SOSTDC1 in TC cells

**Fig. 4:**
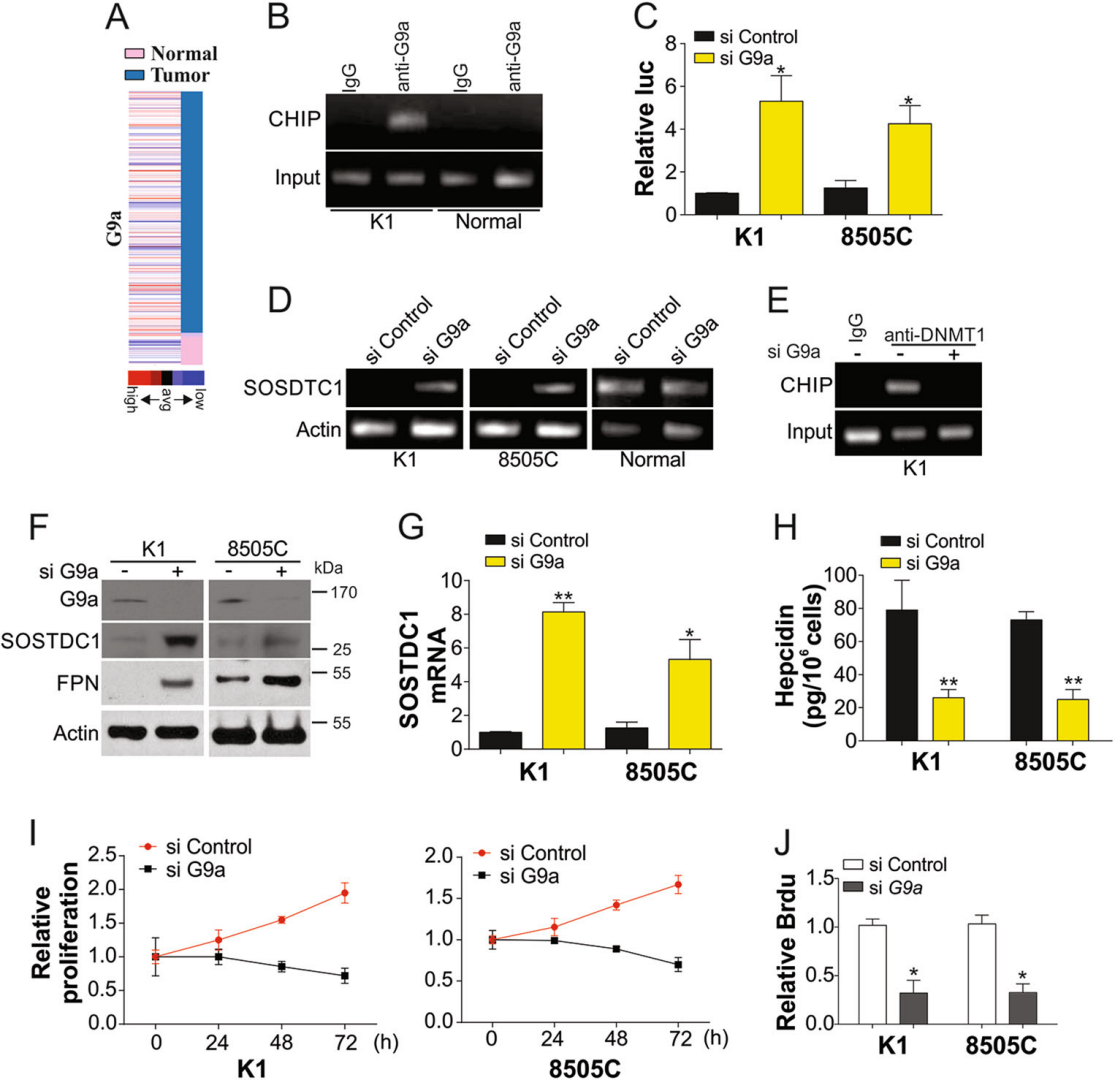
G9a modulated the methylation of SOSTDC1 promoter. **a** G9a expression in TC from TCGA database. **b** Interaction between SOSTDC1 promoter and G9a in K1 cells and Nthy-ori 3-1 cells. **c** The SOSTDC1 luciferase reporter activity in K1 and 8505C cells transfected with control or G9a siRNAs. **d** K1, 8505C, and normal Nthy-ori 3-1 cells transfected with control or G9a siRNAs for 72 h, and analysis of promoter methylation of SOSTDC1. **e** Interaction between SOSTDC1 promoter and DNMT1 in K1 cells with or without G9a siRNA. **f** Proteins expression in K1 and 8505C cells transfected with control or G9a siRNAs for 72 h. **g** mRNA expression of SOSTDC1 in K1 and 8505C cells transfected with control or G9a siRNAs for 72 h. **h** Secretion of hepcidin in K1 and 8505C cells transfected with control or G9a siRNAs for 72 h. **i** The proliferation of K1 and 8505C cells transfected with control or G9a siRNAs for 72 h was analyzed by MTT assay. **j** The proliferation of K1 and 8505C cells transfected with control or G9a siRNAs for 72 h was analyzed by BrdU assay. Each experiment was repeated for three times. *P < 0.05; **P < 0.01

G9a, a H3K9 methyltransferase, is upregulated in many types of human cancers^22^. Therefore, we evaluated whether SOSTDC1 promoter methylation is correlated with G9a expression. G9a was found to be highly expressed in TCs according to the TGCA database (Fig. 4a). This suggested that G9a may be involved in the downregulation of SOSTDC1. We analyzed the binding ability of G9a to the SOSTDC1 promoter via CHIP. As shown in Fig. 4b, G9a exhibited higher ability to bind the SOSTDC1 promoter in K1 TC cells than that in normal thyroid cells. Furthermore, depletion of G9a by siRNA in K1 cells also enhanced luciferase reporter activity of the SOSTDC1 promoter (Fig. 4c). We therefore investigated whether G9a exerts an effect on SOSTDC1 promoter methylation. Results indicated that without G9a, SOSTDC1 promoter methylation was significantly reduced in TC cells, but not in normal thyroid cells (Fig. 4d). Depletion of G9a abandoned the binding of DNMT1, the DNA methyltransferase, with SOSDTC1 promoter (Fig. 4e). Furthermore, absence of G9a significantly enhanced the expression of SOSTDC1 at the protein (Fig. 4f) and mRNA levels (Fig. 4g). Recovery of SOSTDC1 expression by G9a siRNA also enhanced FPN expression (Fig. 4f) and limited the secretion of hepcidin (Fig. 4h). Consistent with previous reports, knockdown of G9a suppressed the proliferation of TC cells (Fig. 4i, j). Collectively, our data suggested that G9a contributes to SOSTDC1 promoter methylation, thereby enhancing hepcidin secretion and promoting TC cell growth.

### G9a is recruited to the SOSTDC1 promoter by E4BP4

**Fig. 5:**
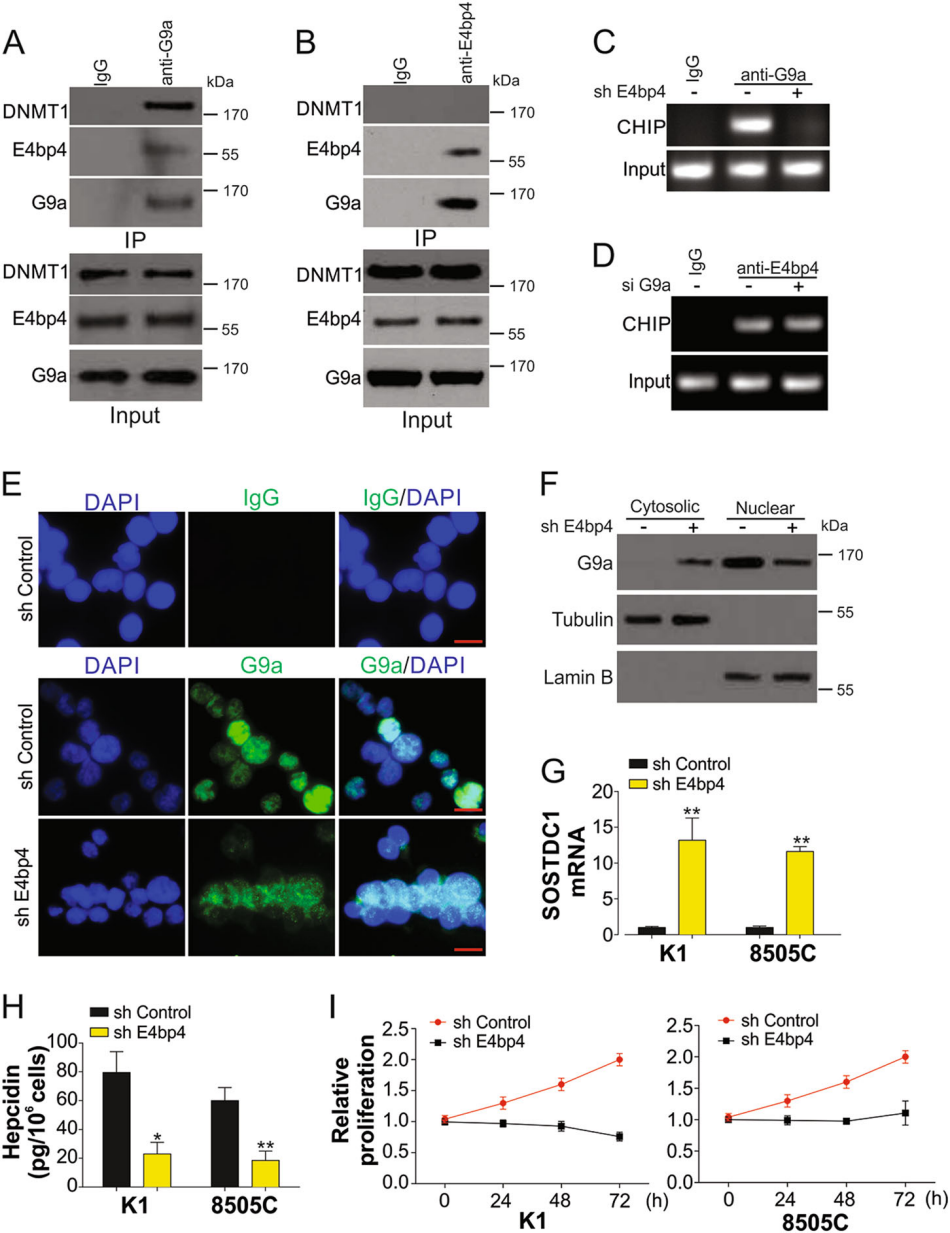
E4BP4 recruits G9a into SOSTDC1 promoter in TC cells. a Interaction of G9a, DNMT1 and E4BP4 analyzed by immunoprecipitation (IP) with anti-G9a antibody. **b** Interaction of G9a, DNMT1, and E4BP4 analyzed by immunoprecipitation (IP) with anti-E4BP4 antibody. **c** The binding of G9a and SOSTDC1 promoter in K1 cells transfected by control or E4BP4 shRNAs. **d** The binding of E4BP4 and SOSTDC1 promoter in K1 cells transfected by control or G9a siRNAs. **e** The immunofluorescence of G9a in K1 cells transfected by control or E4BP4 shRNAs. The IgG staining was used as negative control. Scale bar, 10 µm. **f** The western blot of G9a in cytosolic and nuclear fraction of K1 cells transfected with control or E4BP4 shRNAs. **g** mRNA expression of SOSTDC1 in K1 and 8505 C cells transfected by control or E4BP4 shRNAs. **h** Secretion of hepcidin in K1 and 8505C cells transfected by control or E4BP4 shRNAs. **i** The proliferation of K1 and 8505C cells transfected by control or E4BP4 shRNAs. Each experiment was repeated for three times. *P < 0.05; **P < 0.01

G9a can selectively bind to the SOSTDC1 promoter in different cells (Fig. 4B), suggesting that an underlying mechanism exists for the recruitment of G9a to the SOSTDC1 promoter. E4BP4 is an epigenetic repressor of SOSTDC1^23^, and can interact with G9a in breast cancer^24^. We therefore investigated how E4BP4 affects SOSTDC1 methylation via G9a. Immunoprecipitation analysis revealed that G9a and E4BP4 interact with each other in K1 TC cells (Fig. 5a, b). However, only G9a could interact with DNMT1, but not E4BP4 (Fig. 5a, b), indicating that E4BP4 need G9a to recruit DNMT1 into SOSTDC1 promoter. Depletion of E4BP4 affected the binding of G9a to the SOSTDC1 promoter (Fig. 5c, Fig. S1A). However, G9a inhibition did not (Fig. 5d, Fig. S1B). Furthermore, absence of E4BP4 also affected the nuclear translocation of G9a (Fig. 5e, f), suggesting that recruitment of G9a to the SOSTDC1 promoter is dependent on E4BP4. Consistent with previous results, silencing of E4BP4 in TC cells also rescued mRNA expression of SOSTDC1 (Fig. 5g), suppressed the secretion of hepcidin, and reduced cell proliferation (Fig. 5h, i, Fig. S1C). These data indicated that E4BP4 facilitates binding of G9a to the SOSTDC1 promoter.

### Depletion of E4BP4 inhibits tumor growth in nude mice

**Fig. 6:**
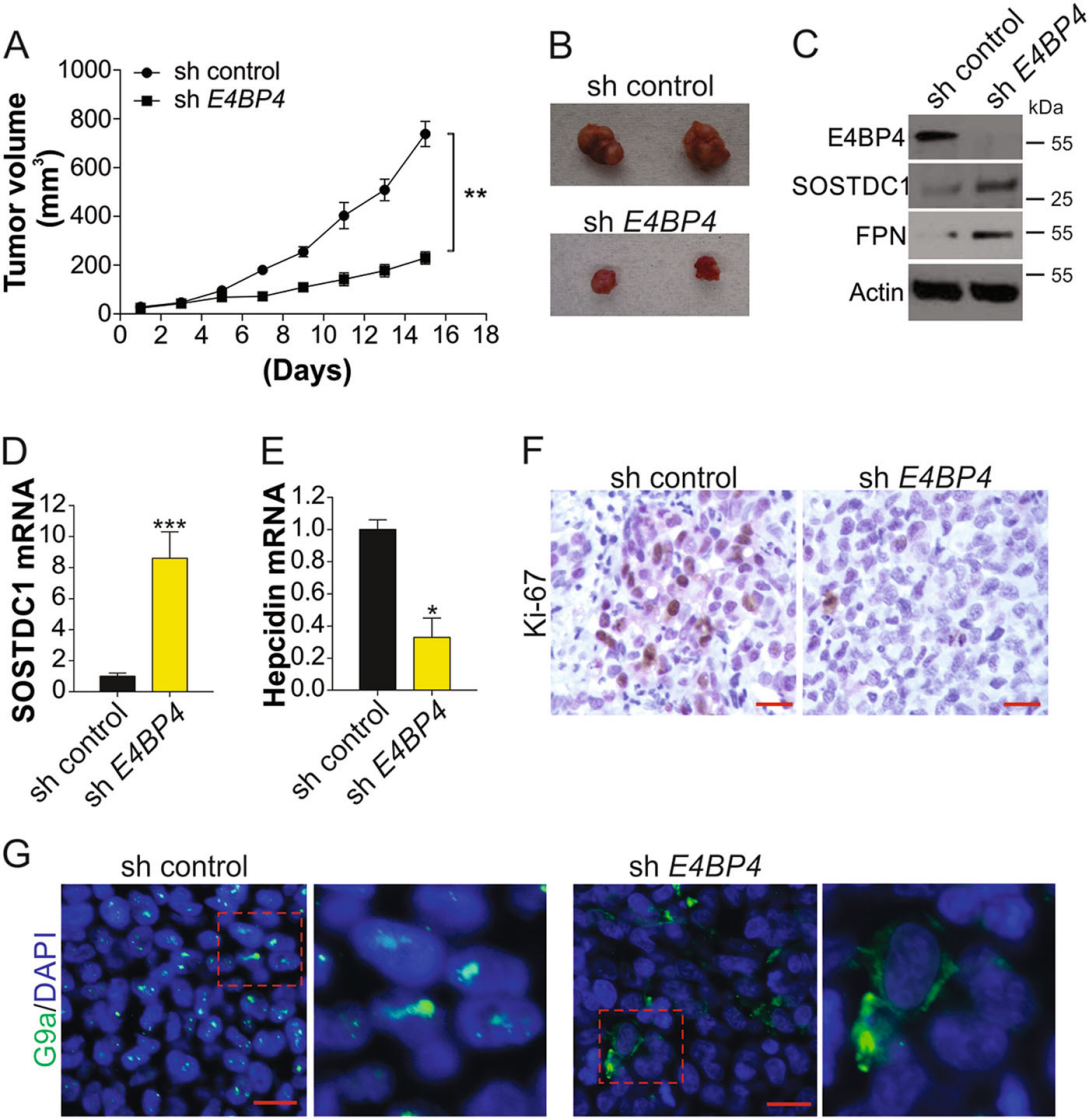
Absence of E4BP4 limits the TC proliferation in vivo. **a** 1 × 10^6^ K1 cells stably transfected with control or E4BP4 shRNA was injected into nude mice to generate xenograft tumors. The growth of tumor was shown. **b** The representative tumors in control or E4BP4 shRNA-transfected group. **c** The expression of indicated proteins in pool samples from control or E4BP4 shRNA-transfected tumors. **d** The mRNA level of SOSTDC1 in control or E4BP4 shRNA-transfected tumors. **e** The hepcidin mRNA in control or E4BP4 shRNA-transfected tumors. **f** The immunochemistry staining of Ki-67 in control or E4BP4 shRNA-transfected tumors. Scale bar, 20 µm. **g** The immunofluorescence staining of G9a in control or E4BP4 shRNA-transfected tumors. Scale bar, 10 µm. Each experiment was repeated for three times. *P < 0.05, ***P < 0.001

E4BP4 shRNA-transfected TC cells were implanted in BALB/c nude mice. Tumor growth was measured every other day after tumors reached a volume of 50 mm^3^. Tumor size was significantly smaller in the E4BP4-knockdown tumors as than in the control groups at 15 days after implantation (Fig. 6a, b). E4BP4 silencing also enhanced SOSTDC1 protein and mRNA expression (Fig. 6c, d), increased the expression of FPN (Fig. 6c), and suppressed hepcidin mRNA expression (Fig. 6e). Moreover, E4BP4 depletion resulted in suppression of Ki-67 (Fig. 6f), further confirming that cancer proliferation was repressed in E4BP4 knockdown tumors. Consistent with the in vitro data, G9a nuclear translocation was also inhibited in E4BP4 knockdown tumors (Fig. 6g). Therefore, E4BP4 can promote TC growth in vivo, and could be a novel therapeutic target in TC.

## Discussion

**Fig. 7:**
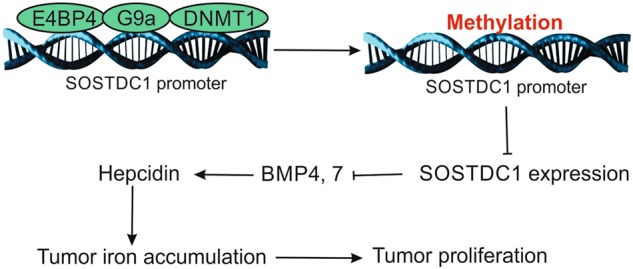
**A model of action for E4BP4 promoting TC cell proliferation.**

Iron is an important factor in cancer development^6^. In various types of cancers, proteins that are essential to biological events require iron for their function^10^. Unlike the majority of normal cells, cancer cells show diversified requirement in terms of protein activity and concentration, both of which may be regulated by iron. In this study, we first found that intracellular iron content is controlled by a small antimicrobial peptide, hepcidin, which could promote the proliferation of TC cells. Our data demonstrated significant upregulation of hepcidin in cancer cells obtained from the serum of patients with TC. We determined that secretion of hepcidin is modulated by SOSTDC1, an agonist of BMP4/7. Furthermore, E4BP4 is the upstream regulator of SOSTDC1, which could recruit the G9a and DNMT1 to the SOSTDC1 promoter. This results in promoter hypermethylation, which eventually leads to inhibition of SOSTDC1 expression to release hepcidin (Fig. 7). We have reported here a general signaling pathway for hepcidin regulation in TC, and these data provide multiple potential therapeutic targets for drug development.

Iron homeostasis is tightly regulated by the hepcidin–FPN axis^25,26^, in which hepcidin is critical to the control of iron flow^25,27^. Hepcidin activation is mainly triggered by degradation of FPN, and elevates intracellular iron levels^8^. No study has examined changes in hepcidin levels in TC, although its increase has been reported in some cancers, such as myeloma^9,12,28^. Our data confirmed increased hepcidin expression in TC and showed that cell proliferation was significantly repressed by hepcidin; serum hepcidin can also affect iron intake in tumor cells, and deletion of hepcidin leads to increased iron export from tumor cells, thereby inhibiting the growth of cancer cells.

IL-6 and BMPs have been found to regulate hepcidin secretion in various cancers, including prostate cancer^12^ and breast cancer^13^. In this study, BMP4/7 exhibited stronger ability to control systemic hepcidin synthesis in TC than did IL-6. Similarly, it was reported that BMP4 expression is involved in crucial biological events in tumors, such as invasion^29^. Therefore, the BMP-antagonist SOSTDC1 can control hepcidin synthesis in TC. BMP antagonists are proteins that regulate the activity of BMP through binding to BMP, thus blocking its interactions with its receptors^30^. SOSTDC1, a BMP antagonist, can competitively bind to BMP2, BMP4, and BMP7, and SOSTDC1 expression is decreased in some cancers^14,31,32^. These reports suggest that SOSTDC1 may exert inhibitory effects on cancer. In this study, we found that SOSTDC1 is downregulated in TC and that it suppresses proliferation of cancer cells. In addition, SOSTDC1 downregulation in TC was associated with methylation of its promoter. In the presence of 5′-aza-dC, which demethylates CpGs in the SOSTDC1 promoter, expression of SOSTDC1 was recovered. In addition, it has been established that promoter methylation may affect the expression of tumor suppressor genes in cancers^33^. It has recently been reported that epigenetic modification results in downregulation of SOSTDC1 in cancer^12^. Furthermore, it was observed that due to methylation, both the activity and expression of SOSTDC1 are suppressed^16^. Hence, epigenetic modification is more likely to function as a suppressor of SOSTDC1 expression in TC. In vitro experiments also revealed the downregulation of SOSTDC1 with promoter hypermethylation, indicating that SOSTDC1 may be a key player in TC.

Our experimental results indicated that the SOSTDC1 promoter is methylated by DNMT1, which is dependent its interaction with G9a in TC cell lines. G9a was originally found to mediate the methylation of H3K9 and H3K27^34,35^; however, several studies also reported that G9a can directly interact with DNMT1 and lead to DNA methylation^36,37^. G9a is highly expressed in many cancers, including human bladder and claudin-low breast cancer^22,38^. Dysregulation of G9a in cancers suggests that it may be a viable therapeutic target^39^. In addition, association between a histone methyltransferase and cellular iron metabolism has been reported^40^. Overexpression of G9a results in suppression of hephaestin and leads to iron accumulation in breast cancer cells, which stimulates cell growth^40^. Here, G9a expression is associated with increased intracellular iron levels in TC cells, and it likely functions via suppression of SOSTDC1 and release of hepcidin. Our results propose a new function for G9a in modulating iron homeostasis during TC development.

Functionality of G9a is dependent on E4BP4. E4BP4 regulates transcriptional modification and is widely expressed in normal cells^41^. High E4BP4 expression is associated with cancers with poor prognosis^42^. As a transcriptional modulator, E4BP4 can inhibit the activity of FOXO1^42^ and is also involved in SOSTDC1 downregulation in breast cancer cells;^23^ its mechanisms of action are currently unknown. It was reported that E4BP4 represses *Fgf21* expression through its interaction with G9a, which disrupts its circadian oscillations in cultured hepatocytes^24^. In this study, we found that E4BP4 suppressed SOSTDC1 by recruiting G9a to its promoter, which methylates the SOSTDC1 promoter to suppress its expression. However, the corresponding mechanisms have not been clarified.

The findings of this study improve the general understanding of the regulation of hepcidin in TC cells, and confirm its role as a major modulator of iron homeostasis in cells. Based on the results of this study, we hypothesize that regulation of hepcidin involves E4BP4, G9a, and SOSTDC1. This is the first report demonstrating that epigenetic modification is critical to the regulation of hepcidin. Future studies are expected to uncover how E4BP4, G9a, and SOSTDC1 function in iron homeostasis. Our molecular model reveals the epigenetic regulation of tumor growth through manipulation of cellular iron homeostasis. These signaling molecules have the potential to be novel therapeutic targets in TC.

## Materials and methods

### Cell culture

BCPAP, TPC1, K1, 8505 C, and Nthy-ori 3-1 cell lines obtained from ATCC (American Type Culture Collection, VA, USA) were cultured in the RPMI-1640 medium. K1 cells in DMEM were mixed with nutrient mixture F-12 in a volume ratio of 1:1.

### Clinical specimens

Samples were collected from 48 patients with sporadic TC and 38 age-matched healthy subjects in RenJi Hospital. Written informed consents were obtained from all subjects. The average ages of patients and healthy subjects were 46.25 ± 7.44 and 46.07 ± 3.98 years, respectively, and diagnoses were made according to the relevant criteria.

### Determination of intracellular iron concentration

Intracellular iron concentration was determined according to an established method^17^. In brief, cells were incubated with CA-AM (Sigma) for 15 min at 37 °C. Then the cells were washed with 2 x  PBS, and were randomly assigned to two treatment groups: one group received a 1 h-treatment of DFO (Sigma) at 37 °C, while the other group received no treatment. Activation and measurement of FCM were performed at 488 nm and 525 nm, respectively, and intracellular iron concentration was calculated by the corresponding formula.

### Protein assay

Harvested cells and frozen tissues were treated with RIPA lysis buffer (Solarbio, China) for protein extraction. Protein lysate was loaded for SDS-PAGE, and was transferred from the gel to the membrane. Unspecific binding was blocked with 5% skimmed milk. Proteins were incubated with β-actin (Santa Cruz), tubulin, FPN (Sigma), DNMT1, SOSTDC1 (Abcam), Lamin B, p-SMAD, SMAD, G9a (Cell signaling), and E4BP4 (Abcam) antibodies at 4 °C overnight. Membranes were then incubated for 1 h with secondary antibodies. Protein detection was performed with the ECL substrate (Millipore, USA), and β-actin was used as the internal control. ELISA kits (R&D Systems) were used to detect levels of BMP4, BMP7, IL-6, and hepcidin.

### Vectors, siRNA, and shRNA transfection

The SOSTDC1 construct was prepared using pcDNA3.1 carrying PCR-amplified SOSTDC1 cDNA. The siRNA for G9a was obtained from ThermoFisher (AM16708). Transfection of plasmid or siRNA was performed by using Lipofectamine reagent (Invitrogen; ThermoFisher). Lentiviral shRNA vector targeting *E4BP4* was purchased from Sigma (NM_005384.1-1746s1c1). Control lentiviral shRNA vector was purchased from Addgene (#10879). Lentiviruses were generated with package plasmids, pMD2.G (VSVG), pMDLg/pRRE, and pRSV-REV (Addgene), together with E4BP4 or control shRNA vectors. The generated lentiviruses were then used to transform K1 and 8505 C cells; successful clones were identified using puromycin (5 ng/mL).

### Quantitative reverse transcription PCR (qRT-PCR)

Total RNA was used for quantification of relevant mRNAs. cDNAs were prepared from extracted RNAs using β-actin as the internal control. qRT-PCR was performed using the following primer sequences: SOSTDC1 primer sense 5′-GCCTGCAAGTGCAAGAGGTA-3′ and antisense 5′-TGCTCTCAAAGTTGTGACTGGA-3′, hepcidin primer sense 5′-CCTGACCAGTGGCTCTGTTT-3′ and antisense 5’-CACATCCCACACTTTGATCG-3′, and β-actin primer sense 5′-CACCAACTGGGACGACAT-3′ and antisense 5′-ACAGCCTGGATAGCAACG-3′.

### 5-Aza-2′-deoxycytidine treatment

Promoter methylation in genomic DNA of normal and TC cell lines was detected as follows: cells were inoculated and subject to culture in medium containing 2 µM 5′-aza-dC (Sigma) for 96 h with medium changes every 24 h. Methylation of SOSTDC1 promoter was analyzed via MS-PCR using the following primers: forward: 5′-TTTTTTAAATGAATAGTGATGTATTTTTGT-3′ and reverse: 5′-ATCACTTATCTATAAACCAACACA-3′. PCR conditions were adjusted as follows: 95 °C for 15 min; 94 °C for 1 min, 60 °C for 40 s, and 72 °C for 45 s (38 cycles); 72 °C for 5 min.

### MTT assay

Cell viability was determined by the MTT assay. Cells were cultured in medium supplemented with 5 mg/mL MTT until appearance of precipitation. The medium was then replaced by 75 mL DMSO, and cells were cultured for 2 h in the dark. Absorbance was measured at 490 nm.

### BrdU assay

TC cells after treatment were pulsed with 5-bromo-2-deoxyuridine (BrdU) for an additional 8 h. Cell proliferation was determined by BrdU incorporation assay according to the manufacturer’s instructions (Roche Diagnostics GmbH, Roche Applied Science, Germany). The absorbance at 450 nm was detected.

### Luciferase reporter assay

To analyze SOSTDC1 promoter activity, the full-length SOSTDC1 promoter regions were cloned into the reporter vector (Promega Corporation, USA). K1 cells were transfected with either vectors containing the SOSTDC1 promoter or control vectors. Luciferase activities were detected three times by dual-luciferase assay system (Promega).

### Chromatin immunoprecipitation (ChIP) and Co-IP

In this study, ChIP was used for detecting the binding status of the SOSTDC1 promoter^18^. For interactions between G9a, DNMT1, and E4BP4, the supernatant of K1 cell extracts was incubated with anti-G9a or anti-E4BP4 antibodies on a shaker overnight at 4 °C. The extracts were then incubated with corresponding beads for another 4 h. Bead suspensions were prepared and used for SDS-PAGE, and immunoblot detection was performed using anti-G9a, anti-DNMT1, or anti-E4BP4 antibodies.

### Nude mouse xenograft model

Nude mice were housed at 28 °C in a specific-pathogen-free room. All procedures were performed according to the guidelines of the Experimental Ethics Committee of RenJi Hospital, Shanghai JiaoTong University. K1 cells successfully transfected with control or *E4BP4* shRNA were injected into nude mice (6 weeks). When tumor size reached ~50 mm^3^, tumor volume was measured every other day for 15 days. The dissected tumors were fixed with 10% formalin before they were embedded within paraffin, or homogenized to extract proteins for western blot and mRNA for RT-PCR. Tumor slices embedded with paraffin were subjected to immunofluorescence staining and immunochemical staining.

### Statistical analyses

Intergroup comparison was performed by unpaired *t*-tests, and statistical significance was set as *p* *<* 0.05.

## Electronic supplementary material

supplementary figure legends

S1

S2
