# Supplementary material for: E4BP4 promotes thyroid cancer proliferation by modulating iron homeostasis through repression of hepcidin
Source: Cell Death Dis. 2018 Sep 24;9(10):987. doi: 10.1038/s41419-018-1001-3 (PMC6155336; doi:10.1038/s41419-018-1001-3)
Supplement: Supplementary file 1 — supplementary figure legends [file 41419_2018_1001_MOESM1_ESM.doc]

**Fig. S1 E4BP4 mediates G9a binding with SOSTDC1 promoter and TC cell proliferation.** (A) The binding of G9a and SOSTDC1 promoter in K1 cells transfected by control or E4BP4 shRNAs was quantified by real-time PCR. (B) The binding of E4BP4 and SOSTDC1 promoter in K1 cells transfected by control or G9a siRNAs was quantified by real-time PCR. (C) The proliferation of K1 and 8505C cells transfected by control or E4BP4 shRNAs was analyzed by Brdu assay. Each experiment was repeated for 3 times. *P < 0.05, ** P<0.01.

**Fig. S2 The primary data for Fig. 5C.**
