# Supplementary figures and images for: E4BP4 promotes thyroid cancer proliferation by modulating iron homeostasis through repression of hepcidin
Source: Cell Death Dis. 2018 Sep 24;9(10):987. doi: 10.1038/s41419-018-1001-3 (PMC6155336; doi:10.1038/s41419-018-1001-3)

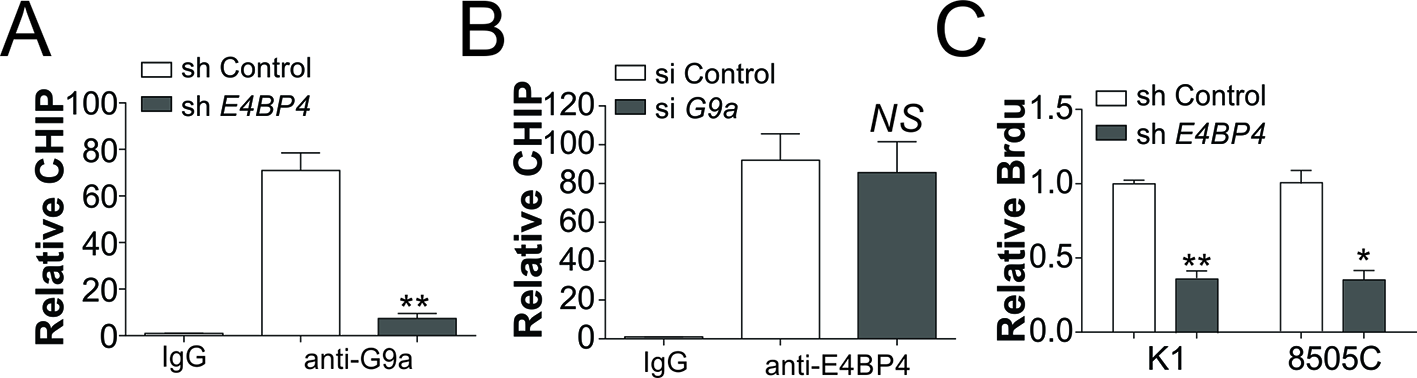

Supplement: Supplementary file 2 — S1 [file 41419_2018_1001_MOESM2_ESM.tif]

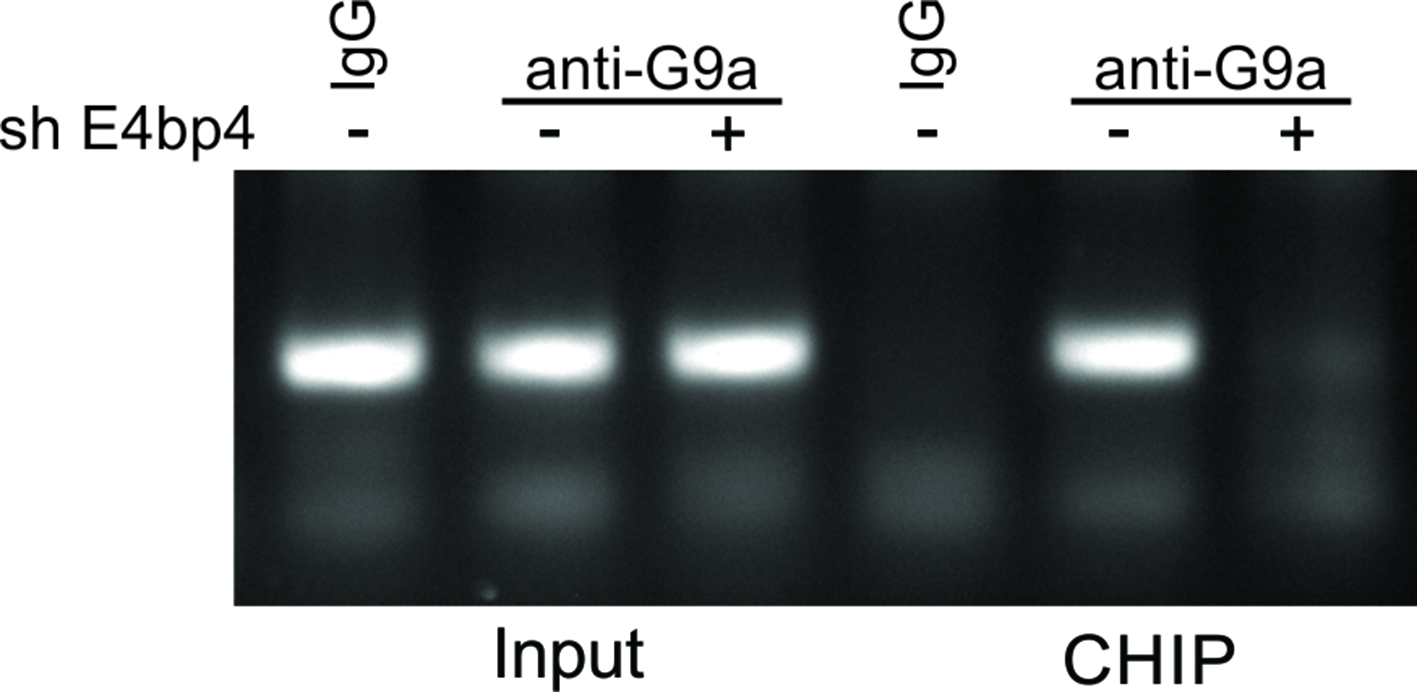

Supplement: Supplementary file 3 — S2 [file 41419_2018_1001_MOESM3_ESM.tif]
